# Glycemic control and correlates in a group of sub Saharan type 1 diabetes adolescents

**DOI:** 10.1186/s13104-019-4054-1

**Published:** 2019-01-22

**Authors:** Cathy Djonou, Aurel T. Tankeu, Mesmin Y. Dehayem, Daryl N. Tcheutchoua, Jean Claude Mbanya, Eugene Sobngwi

**Affiliations:** 1 Faculty of Medicine and Biomedical Sciences, University of Yaounde I, Yaoundé, Cameroon; 2 National Obesity Center, Yaounde Central Hospital, Yaoundé, Cameroon

**Keywords:** Type 1 diabetes, Africa, Glycemic control

## Abstract

**Objectives:**

This study aims to describe the prevalence of glycemic control and related factors in a population of Sub-Saharan African T1D patients. We carried out a cross-sectional study including children and adolescents from seven different centers of the Changing Diabetes in Children (CDiC) program. All children enrolled in the program where recruited after parental consent. Diabetes history, daily practice anthropometrics parameters and HbA1c were assessed for each participant.

**Results:**

We enrolled 95 children adolescents, aged from 06 to 19 years. The mean HbA1c was 9.2 ± 2.5% and 67.4% of participant had poor glycemic control. There was an association between study level of the patients (p = 0.03), healthy eating habits (p < 0.001), diabetes duration (p < 0.001) and level of glycemic control on univariate analysis. On multivariate analysis, diabetes diagnosed for more than 2 years was associated to a good control compared to those with diagnosis that is more recent. Glycemic control of adolescents with type1 diabetes remain very poor in Cameroon despite the implementation of free diabetes care through the program CDiC.

## Introduction

Type 1 diabetes mellitus (T1DM) is a chronic disease with a high prevalence and a growing concern worldwide [1]. It represents the main metabolic disease and one of the most common pediatric endocrine illness [2] affecting approximately 78,000 children, with 70,000 new cases diagnosed each year [3]. This condition usually affects children and adolescents leading to short- and long-term complications resulting in a high morbidity [4]. About 80% of diabetes deaths occur in low- and-middle income countries [1] and are mainly related to poor glycemic control [5, 6]. There are evidences that good glycemic control or even near-normal glycemic control can prevent target organ damage [7]. However, despite continuous improvement means and supported protocols, majority of patients fail to achieve good glycemic control. According to the American Diabetes Association, only 21% of adolescents with type 1 diabetes (T1D) meet glycemic goals [8]. This situation is alarming on African continent due to lack of financial and human resources. This failure to achieve adequate glycemic control can have substantial consequences on long-term health outcomes and contribute to the development of early complications in multiple organ systems [6]. Therefore, it is important to identify and describe the main correlates of glycemic control in sub Saharan type 1 diabetes adolescents [8]. However, data on this topic are still scare. In order to fill this gap knowledge, we carried out this study, which aimed to describe the glycemic control, and their main correlates in a group of sub Saharan African type 1 diabetic children and adolescents.

## Main text

### Methods

#### Ethical considerations

the institutional ethical Committee of the Faculty of Medicine and Biomedical Sciences of the University of Yaoundé I approved this study. This latter was conducted in accordance with the guidelines of the Helsinki Declaration.

#### Study setting, design and period

We carried out a cross-sectional study from September 2012 to March 2013 in different centres of diabetic children’s care belonging to the Changing Diabetes in Children (CDiC) program. CDiC is a program established as a public–private partnership which aim to improve delivery of diabetes care to children and adolescents with type 1 diabetes in resource-poor settings. All children and adolescents attending those centres are provided with insulin at no cost. In addition, they are provided with glucose monitors, strips and diaries for self-monitoring and recording of blood glucose at home. They are encouraged to monitor their glucose three times per day at specified times and to record the readings in their diaries.

HbA1c is measured for all patients every 3 months, in whole blood with an automated nonporous ion-exchange high-performance liquid chromatography system. Patients and guardians are also given diabetes education and advice on appropriate nutrition.

#### Recruitment of participants

All children and adolescents from 6 to 19 years old, leaving with type 1 diabetes, followed in centres of CDiC for at least 1 year, were eligible for inclusion in the study if the parent/guardian along with the adolescent provided a written consent. We included all the children and the teenagers filling the criteria of inclusion who accepted to participate to the study.

#### Data collection

We used a structured questionnaire including sociodemographic data, diabetes history, insulin regimen and dosage, diabetes related practices including:

- Insulin adherence: This was determined by the number of insulin doses missed in an ordinary week and was graded as follows: *Good *= no missed doses; *Poor *= 1 or more missed doses.
- Dietary adherence: This was assessed by use of 8 elements concerning diet of the Diabetes Self-Management Profile (DSMP) questionnaire. The DSMP is a 25-items structured interview that assesses adherence to diabetes self-management. Nine questions concern the adherence to dietary recommendation, 1 was omitted because it was not applicable to our patient population.
- Blood glucose monitoring (BGM) adherence.

### Data analysis

Data entry was done using Epi Info software version 3.5.1 and analysed using SPSS 18.0 and STATA 9. Data are reported as mean ± standard deviation for continuous variables and proportions/frequencies for categorical variables. The Chi square test was used to test the association between HbA1c and the characteristics of the participants. Binary logistic regression served for multivariate analysis. Statistical significance was set at p = 0.05.

### Results

#### General characteristics and glycemic control in our sample population

We included 95 children and adolescents (45 males) aged from 6 to 19 years with a mean duration of diabetes 4.1 ± 2.9 years. The mean daily insulin dose was 0.79 ± 0.32 U/kg/day and 68.4% of participants were taking at least 03 injections/day. The mean HbA1c was 9.2% ± 2.5. 67.4% of participants have glycosylated hemoglobin above glycemic targets for age versus 32.6% of well-controlled patients. Sixty percent of well-controlled patients were girls. The Table 1 summarized the demographic characteristics of the study population. The study level of patients was associated to the glycemic control with 66.7% of participants who reached the glycemic target were high school students.

**Table 1:** Demographic characteristics of the study population

Patients characteristics	Frequency	Percentage
Age (years)		
6–12	10	10.5
13–19	85	89.5
Sex		
Male	50	52.6
Female	45	47.4
Study level of the child		
None/primary school	30	31.5
Secondary school	60	63.2
University	5	5.3
Hypoglycemia during last 12 months		
Yes	30	31.5
No	65	68.5
Number of hypoglycemia episodes		
< 5	24	68.6
> 5	11	31.4
Children surroundings		
Living with one parent	19	20
Living with both parents	51	53.7
Not living with parents parents	25	26.3
Diabetes duration (years)		
< 2	22	24.5
[2–4]	33	36.2
[4–7]	23	21.3
7 +	17	18.1
Number of insulin injection		
2 injections/day	30	31.9
3 injections/day	63	66.3
> 3 injections/day	2	2.1
Frequency of daily glucose control		
< 2	8	8.6
2–3	48	51.6
> 3	37	39.8
Diet counselling		
Yes	75	78.9
No	20	21.1
Mean glycated hemoglobin per age range/per sex		
Age range		
6–12	90.51 ± 3.84	
13–19	9.28 ± 4.58	
Sex		
Male	9.41 ± 4.2	
Female	9.19 ± 4.84	
Glycemic control per age range/per sex		
Good	31	67.4
Poor	64	32.6

#### Factors associated to poor glycemic control

On unadjusted univariate analysis (Table 2), three parameters were associated to glycemic control in our population sample. The study level of the patients (p = 0.03), healthy eating habits (p < 0.001) as well as duration of type 1 diabetes (p < 0.001). When adjusted for confusion factors only diabetes duration was independently associated to glycemic control (Table 3). Thus, patients diagnosed for more than 2 years presented a better glycemic control than those with diagnosis that is more recent.

**Table 2:** Univariate analysis

Variable	Poor control	Good control	OR (95% CI)	p-value
Age range (years)				
6–12	8	2	–	
13–19	56	29	1.06 (0.91–1.24)	0.36
Sex				
Male	39	11	–	
Female	26	19	1.47 (0.91–2.8)	0.1
Study level				
Before college	64	26	–	
College	0	5	0.7 (0.55–0.79)	0.03*
Hypoglycemia during last 12 months				
Yes	22	08	–	
No	42	23	0.51 (0.21–1.23)	0.08
Child living with both parents				
Yes	32	14	–	
No	26	11	0.98 (0.58–1.66)	0.57
Diabetes duration (years)				
< 2	53	22	–	
> 2	10	9	0.43 (0.23–0.79)	< 0.001*
Eating habits				
Good	4	10	–	
Poor	60	21	1.3 (1.12–2.6)	< 0.001*
Frequency of daily glucose control				
Once	5	3	–	
> 2/day	59	28	0.96 (0.81–1.13)	0.46
Insulin regimen				
2 injections/day	21	9	–	
> 2 injections/day	43	22	0.92 (0.65–1.33)	0.43
Physical activity				
Sedentary	20	12	–	
Active	44	19	0.91 (0.46–1.78)	0.49

* for p < 0.05

**Table 3:** Multivariate analysis

Variable	Poor control	Good control	Univariate analysis		Multivariate analysis	
			OR (95% CI)	p-value	OR (95% CI)	p-value
Sex						
Male	39	11	–		–	
Female	26	19	1.47 (0.91–2.8)	0.1	0.87 (0.19–4.02)	0.86
Study level						
Before college	64	26	–		–	
After college	0	5	0.7 (0.55–0.79)	0.03		0.9
Hypoglycemia during last 12 months						
Yes	22	08	–		–	
No	42	23	0.51 (0.21–1.23)	0.08	1.39 (0.26–7.27)	0.69
Diabetes duration (years)						
< 2	53	22	–		–	
> 2	10	9	0.43 (0.23–0.79)	< 0.001	0.07 (0.01–0.43)	0.004*
Eating habits						
Good	4	10	–		–	
Poor	60	21	1.3 (1.12–2.6)	< 0.001	1.69 (0.23–11.78)	0.6

* for p < 0.05

### Discussion

We carried out this cross-sectional study in order to evaluate the glycemic control in type 1 diabetes adolescents and their determinants. We found very high proportion of uncontrolled type 1 diabetes patients in our population sample with two third of patients that did not reach the glycemic targets for age. The most important determinant of poor glycemic control was duration of diabetes. The determinant of poor glycemic control were the low study level of the patient, unhealthy eating habits and short duration of diabetes.

These findings are consistent with reports of Samahy et al. in Egypt who found about 71% of uncontrolled type 1 diabetes in their population sample [9]. But this is in sharp contrast with findings reported in developed countries. For instance, in United States, only 17% of T1D were uncontrolled [10]. This important difference between developed and developing countries mostly reflects the impact of economic level on diabetes management since diabetes represent a huge economic burden for the families [11]. In low-and-middle income countries where type 1 diabetes related costs are supported by the family of patients, the management of T1D is usually suboptimal resulting in a poor metabolic control and higher level of complications [12]. Therefore, improvement in type 1 diabetes control require an increase of investments in diabetes care in these regions.

Similarly to our findings, Ramchandani et al. also reported in United States, an improvement of glucose control with the transition from high school to college [13]. This stress the importance of patient education in the management of type 1 diabetes. Concerning the role of diet in the managements of T1D, Due et al. found in 2013 in a group of adolescent with type 1 diabetes, an association between eating habits and glycemic control [14]. In the same vein, unhealthy habits was associated with poor glycemic control in our study. This is logical considering that almost all participants were on a conventional insulin protocol with fixed injections usually centered and calibrated on the tree daily main meals. Therefore, any deviation in the diet will modify the glycemia and repetition of such habits will lead to poor glycemic control. Regarding diabetes duration, our study shows that glycemic control improves with diabetes duration in adolescents. This finding, however, is contradictory to findings in T2DM, but it makes sense in our context, especially in adolescents with type 1 diabetes, who have control very closely related to understanding their disease and adopting healthy behaviors. As a result, adolescents diagnosed for a long time have had the time to become familiar with their illness, to go through the psychological phase of denial and to accept their condition and its implications and requirements. They are therefore more likely to bend to the multiple sacrifices necessary to obtain good glycemic control than those recently diagnosed which very often still go through a phase of denial of the disease.

### Conclusion

Glycemic control of T1D adolescents is still poor in our context despite the implementation of large children diabetes care’s program which provide free management to these patients in the country. This poor glycemic control is mainly determined by diabetes duration.

## Limitations

The main limitation of this study is the small sample size. However, this study included all type 1 diabetes adolescents and children of the country identified and enrolled in the CDiC program. Therefore, the study sample is exhaustive and representative of type 1 diabetes patients of the country.

## Abbreviations

T1D: type 1 diabetes
CDiC: Changing Diabetes in Children
HbA1c: glycated haemoglobin
DSMP: Diabetes Self-Management Profile
BGM: blood glucose monitoring
CDC: Centre for Disease Control
